# Short-term effects of the whole-body vibration on the balance and muscle strength of type 2 diabetic patients with peripheral neuropathy: a quasi-randomized-controlled trial study

**DOI:** 10.1186/s40200-015-0173-y

**Published:** 2015-05-23

**Authors:** Amin Kordi Yoosefinejad, Azadeh Shadmehr, Ghloamreza Olyaei, Saeed Talebian, Hossein Bagheri, Mohammad Reza Mohajeri-Tehrani

**Affiliations:** Physical Therapy Department, School of Rehabilitation, Shiraz University of Medical Sciences (SUMS), Chamran Blvd, 1st Abivardi Ave, Shiraz, IR Iran; Physical Therapy Department, Rehabilitation Faculty, Tehran University of Medical Sciences (TUMS), Pich-e- Shemiran, Tehran, IR Iran; Endocrinology and Metabolism Research Center, Endocrinology and Metabolism Clinical Sciences Institute, Tehran University of Medical Sciences, Tehran, Iran

**Keywords:** Type 2 diabetes, Whole body vibration, Neuropathy, Balance, Muscle strength, TUGT

## Abstract

**Background:**

Patients with diabetes type 2 suffer from many complications such as peripheral neuropathy (PN). PN impairs postural stability and muscle strength. Therapeutic exercise may improve functional abilities of diabetic patients but they are unwilling to participate in exercise programs. Whole Body vibration (WBV) is a new somatosensory stimulation which is easy to use and time-efficient. The effects of WBV on balance and strength of diabetic patients had not been studied; therefore the aim of this study was to assess the effects of WBV in type 2 diabetes patients.

**Methods:**

It was a quasi-RCT study performed between March 2011 and February 2013. Twenty patients were randomly assigned into either a whole body vibration group, or a control group. WBV group received vibration (frequency: 30 Hz, amplitude: 2 mm) twice a week for 6 weeks.

Muscle strength, Timed Up & Go Test (TUGT) and Unilateral Stance Test and balance parameters were measured at baseline and after the intervention.

**Results:**

WBV had significantly increased strength of tibialis anterior (*P* = 0.004) and quadriceps muscles (*P* = 0.05) after 6 weeks of training. TUGT time decreased significantly (*P* = 0.001) in the WBV group.

**Conclusions:**

Application of WBV enhanced muscles strength and balance in patients with diabetes type 2-induced peripheral neuropathy. The changes may be due to muscle tuning hypothesis and altered postural control strategies.

**Trial registration:**

IRCT201106156806N1

## Background

Type 2 diabetes is a worldwide disease with several secondary complications. The International Diabetes Federation has reported that the prevalence of diabetes is 9.3 % in the Iranian population aged 20–79 years [1]. It is estimated that the population of diabetics will reach about 300 million by 2025 all round the world with higher prevalence at developing countries [2].

Peripheral neuropathy is a common complication involves more than half of the patients with diabetes [3, 4] accounts for lower extremity somatosensory deficits as well as postural impairments and a high risk of falling [5–7]. Sayer et al. demonstrated that diabetic patients have significantly declined muscle strength and higher odds of impaired physical function in comparison to those without diabetes [8].

Recent studies revealed that balance exercises in conjunction with function-oriented strengthening exercises have the potential to improve the balance and muscle strength in patients with diabetes [9–11]. However, elderly people may not comply with conventional balance and resistance exercises. Whole-Body vibration (WBV) may be an appropriate alternate that augments neuromuscular activation of leg musculature in response to an acute vibratory stimulus [12]. Evidence is emerging that WBV has a profound effect on muscle performance by significantly increasing the strength in patients with varying disabilities [13–16]. WBV improves postural steadiness performance in older population [17–19]. However, there is only one case report study that has evaluated the short term effects of WBV on patients with type 2 diabetes [20]. Therefore the objective of this study was to evaluate the effects of WBV on balance and strength in patients with type 2 diabetes accompanying mild to moderate peripheral neuropathy.

## Methods

**Fig. 1:**
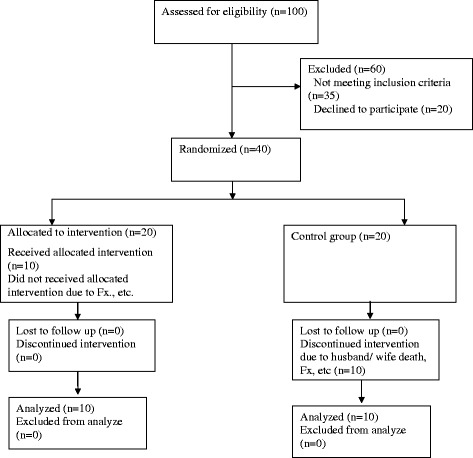
Consort Flow diagram of the participants

This study was a single-blind, single factor pretest-post-test control-group design. The study was approved by the ethics committee of Endocrinology and Metabolism Research Center (EMRC) in accordance with the standards of the Helsinki declaration and guideline of the Iranian Ministry of Health and Medical Education. More than 120 patients were evaluated in Diabetes and Metabolic Diseases center of Tehran University of Medical Sciences between March 2011 and February 2013 and forty clients were selected to participate in our study. (Fig. 1) The patients were diagnosed by endocrinologist as type 2 diabetes. The primary outcome measure of the study was mean velocity of CoP. The secondary outcome measures were the isometric strength of quadriceps and tibialis anterior muscles; anterior-poterior and mediolateral displacements, and total area of displacement. The inclusion criteria were having had the history of Diabetes Mellitus according to the American Diabetes Association (ADA) guideline 2001 [21] or using oral hypoglycemic agent; HbA1C < 8.5 %; Body Mass Index (BMI) between 25 and 35; Michigan Diabetic Neuropathy Score (MDNS) [22, 23] between 13 and 29 (mild to moderate neuropathy) and age between 50 and 70 years. Exclusion criteria were mainly based on contraindications of WBV: epilepsy, cognitive disorders, knee or hip prosthesis, pacemaker and gall or bladder stone. All subjects read participant information sheet and signed an informed consent after being selected for the study.

### Randomization

Block permutation method was applied using a computer based program to assign subjects to either group randomly with block size of two. As we imagined that in this way dropouts would be high, 120 patients were initially evaluated. The groups were matched on age, BMI, and the degree of peripheral neuropathy. Demographic data of the groups is summarized in Table 1. A physical therapist not involved in care or trial was responsible for allocation procedure. The randomization schedule was concealed from all care providers, and other research personnel.

**Table 1:** Demographic data of WBV intervention and control groups

Variable	WBV group (*N* = 10)	Control group (*N* = 10)	*P*-value
age (years)	1.8 ± 57	57 ± 1.5	0.86
gender (F/M)	6/4	6/4	---
Height (cm)	164 ± 3.3	158 ± 3.0	0.22
Weight (Kg)	75 ± 2.0	72 ± 3.5	0.43
BMI (kg.m^−2^)	28.5 ± 1.0	28.9 ± 1.0	0.77
duration of diabetes (years)	11 ± 1.6	12 ± 2.0	0.73
duration of neuropathy (months)	29 ± 6.5	22 ± 8.0	0.33

Data are mean ± SE

### Initial assessment

Initial assessment had three subgroups: Strength measurements; Functional tests, and Balance evaluation. These tests were also randomly performed.

### The isometric strength measurements

Strength was measured both locally and generally. To assess strength locally, two muscles were evaluated: quadriceps femoris and tibialis anterior.

For assessing the isometric strength of quadriceps muscle, subject laid prone on a plinth. A dynamometer (MIE, medical research Ltd., England) was fixed to a frame over the plinth. Trunk and non-dominant limb were fixed to plinth using straps. With dominant knee at 90° flexion, three isometric contractions were performed and the average of three trials was calculated.

To evaluate tibialis anterior muscle isometric strength, subject sat at the edge of the plinth, the non-dominant leg was fixed with strap. The ankle joint of the dominant leg was at the neutral position. The dynamometer was fixed to the frame inferiorly. The subject was asked to perform isometric dorsi flexion. Three trials were performed and the average of three was calculated.

To evaluate general strength, we used a Back-Leg-Chest dynamometer (BASELINE, USA). As can be inferred from the name, this dynamometer evaluates the total strength of trunk, lower extremities, and chest muscles. Subject stood on the plate and with straight elbows and knees, pulled-up the grip bar three times sequentially. General strength was scored as the mean of the three trials.

The strength data were measured in kg.

### Functional tests

We performed two functional tests.

### Timed Up & Go Test (TUGT)

TUGT is a valid test for mobility and dynamic balance [24]. Participants were asked to rise from a chair, walk 3 m to a point on the floor at their usual comfortable safe pace, turn around the point and return to their initial seated position. TUGT was scored as the mean time of three subsequent trials.

### Unilateral Stance Test (UST)

UST is a commonly-used measure of the balance capabilities, and also is a significant predictor of falling [25] in peripheral neuropathy [26]. With the arms folded across the chest, participants stood on the dominant leg and lifted the other limb approximately 5 cm from the medial malleolus of the stance leg. Three trials of UST were performed and then the average time of three was calculated.

### Balance evaluation

To evaluate the balance, eight different positions were selected to perform on the force plate (MIE Medical Research Ltd., Bertec Forceplates, 9090 series, UK). Positions are summarized in Table 2. The sampling frequency was 400 Hz and each position was maintained for 30 s.

**Table 2:** Different positions maintained on plate form to evaluate balance

Position	Eyes	Foam	Standing on one/two legs
1	open	No	two legs
2	open	No	one leg
3	open	Yes	two legs
4	open	Yes	one legs
5	closed	No	two legs
6	closed	No	one leg
7	closed	Yes	two legs
8	closed	Yes	one leg

Each position was held for three times. The patients randomly performed the tests; each position was assigned a number and the patient selected a number. After performing the balance tests, we found that none of the participants could perform the positions number 6 and 8, so the maximum number of performed tests was 18.

### WBV parameters

WBV (Power-Plate, Next Generation, USA) was applied twice a week for 6 weeks (12 sessions). The applied frequency was 30 Hz and the peak-to-peak amplitude was 2 mm. The peak acceleration was calculated as 3.61 g (35.41 m.s^−2^) following the guidelines of the International Society of Musculoskeletal and Neuronal Interactions [27]. The plate was a synchronous type. The application time increased every 2 weeks from 30 s initially to 45 s for 3rd and 4th weeks and to 1 min for two last weeks. The subjects stood barefooted with an equal weight distribution over both feet on the plate while maintaining 30° of knee flexion. All participants were asked to contract the muscles of the lower limbs during exposure to vibration and permitted to bear more weight on their forefoot to reduce resonance and also to dampen the transmitted vibration waves [13]. Participants were not allowed to touch the handle of vibrating plate. No skidding was occurred during the application of WBV [27]. The WBV training process was supervised by an expert physical therapist.

The control group did not receive WBV and also did not participate in any physical activity training which might have affect the results. After 6 weeks, muscle strength, functional tests and the balance were evaluated again in all participants.

### Statistics

Our dependent variables in the study were quadriceps, tibialis anterior, and general muscle strength, functional tests, and balance parameters. The Statistical Package for Social Science version 18 (SPSS Inc, Chicago, IL, USA) was used for the data processing. Having normally distributed according to Kolmogorov-Smironov test, we analyzed our data using parametric statistical methods. Quantitative data were presented as mean and standard deviation and the independent sample *t*-test and ANOVA repeated measure were used for analyzing data. ICC two way mixed was used to analyze different parameters of the data derived from force plate. The effect size of the outcomes was calculated using Cohen’s-d formula. All tests were two-tailed and considered to be statistically significant at P < 0.05.

## Results

Twenty type2 diabetic patients with neuropathy were assigned randomly to WBV intervention and control groups. There were no significant differences statistically between measured variables at the baseline. Baseline measurements are summarized in Table 3.

**Table 3:** Baseline data of all measured variables in WBV intervention and control groups

Variable	WBV intervention group (*n* = 10)	Control group (*n* = 10)	*P*-value
Quadriceps strength (kg)	12.4 ± 2.4	12.5 ± 2.0	0.96
Tibialis Anterior strength (kg)	6.3 ± 0.6	7 ± 1.0	0.59
General strength (kg)	122 ± 22	113 ± 24	0.76
TUGT (sec)	9.3 ± 0.8	9.15 ± 0.4	0.84
UST (sec)	2.8 ± 0.2	3.2 ± 0.6	0.48
ONF-Velocity (cm.s^−1^)	4.5 ± 0.7	4 ± 0.19	0.4
OF- Velocity (cm.s^−1^)	5.1 ± 1.2	3.7 ± 0.09	0.32
CNF-Velocity (cm.s^−1^)	5.4 ± 1.5	4 ± 0.19	0.38
CF-Velocity (cm.s^−1^)	7.3 ± 1.7	4 ± 0.19	0.09

Data are mean ± SE

TUGT, Timed Up & Go Test; UST, Unilateral Stance Test; ONF, Open eye No foam; OF, Open eye with foam; CNF, Closed eye No foam; CF, Closed eye with Foam

### Strength

- Quadriceps muscle strength: quadriceps muscle strength changed from 12.4 ± 2.4 kg and 12.5 ± 2 kg at the baseline to 15.4 ± 2 kg and 12.2 ± 1.6 kg after the study in WBV intervention and control groups respectively. The percentage of changes was significant statistically between groups (*P* = 0.02). The effects size was 0.57.
- Tibialis anterior muscle: tibialis anterior muscle strength changed from 6.3 ± 0.6 kg and 7 ± 1 kg at the baseline to 10.7 ± 1.2 kg and 7 ± 0.8 kg post study in WBV intervention and control groups respectively.
  The percentage of changes was significant statistically between the groups (*P* = 0.004). The effect size of tibialis anterior was 1.13.
- General muscle strength: Using Back-Leg-Chest dynamometer to assess general muscle strength, it changes from 122 ± 22 kg and 112.6 ± 24 kg to 136 ± 20 kg and 118.5 ± 23 kg in WBV intervention and control groups respectively. The percentage of changes was not statistically significant (*P* = 0.48). The effects size was calculated as 0.26.

### Functional tests

#### TUGT

TUGT changed from 9.3 ± 0.8 s and 9.15 ± 0.4 s to 8.5 ± 0.7 s and 9.8 ± 0.3 s in WBV intervention and control groups respectively. Percentage of changes between WBV intervention and control groups showed statistically significant difference (*P* = 0.002). The effect size was 0.83.

#### UST

UST time increased from 2.8 ± 0.2 to 4 ± 0.8 s in WBV intervention group and decreased from 3.2 ± 0.6 to 2.5 ± 0.4 s in control group. There is not any significant difference in percentage of changes between the groups (*P* = 0.22). The effects size was 0.69.

### Balance evaluation

We evaluated four (each position was assessed for three times) out of eight designed positions because only three of the participants in each group could accomplish one foot standing positions. Using ICC two way mixed analyses, mean velocity had a high reliability in all test positions in comparison with other force plate parameters including anterior-posterior and medio-lateral displacement, and area of sway. ICC of mean velocity of all positions is summarized in Table 4.

**Table 4:** ICC of the outcomes

Parameter	*F*-value (19,38)	ICC	*P*-value
mean velocity (ONF)	20.42	0.95	<0.001*
mean velocity (CNF)	23.45	0.95	<0.001*
mean velocity (OF)	42.78	0.97	<0.001*
mean velocity (CF)	50.42	0.98	<0.001*
quadriceps strength	10.07	0.89	<0.001*
tibialis anterior strength	15.00	1.23	<0.001*
general strength	50.30	0.90	<0.001*
TUGT	5.22	0.80	<0.001*
UST	4.30	0.76	<0.001*

Because of high ICCs, we analyzed mean velocity of four conditions. The effects size of the evaluated conditions was as follows: open eye without using foam: 0.39, open eye with foam: 0.24, closed eye without using foam: 0.52, and closed eye with foam: 0.55.

Using ANOVA repeated measure; we observed significant main time effect when the participants closed their eyes and stand on the foam (F1, 18 = 3.9, *P* = 0.04).

**Fig. 2:**
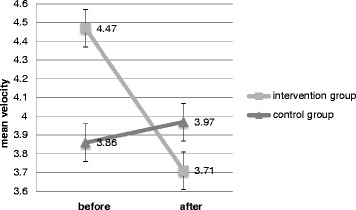
Interaction between time and group effects of mean velocity when Participants kept eyes closed and stood on the foam. Pattern of changes of mean velocity is different pre- and post intervention

Also, there was significant interaction between time and group effects (F1, 18 = 6.96, *P* = 0.01). Therefore, pattern of changes of mean velocity is different pre- and post intervention (Fig. 2).

No attrition was reported. The subjects did not report any adverse effects either during or after the application of WBV and 8 out of 10 subjects in the intervention group were adhered to continue the training sessions after finishing the study.

## Discussion

The aim of our study was to evaluate the effects of a 6-week training program of WBV on the balance and the muscle strength of diabetic patients with neuropathy. To our best knowledge, it is the first study investigating the balance and muscle strength in type 2 diabetic patients with peripheral neuropathy.

### Isometric strength

Tibialis anterior and quadriceps muscles isometric strength confirmed statistically significant improvement in WBV intervention group in comparison to control group. These changes can be attributed to neurogenic adaptations [13]. As vibration waves transmitted to lower extremities, they allowed more activation of the prime movers and better coordinated activation of the muscles, both accounted for a greater force generation. Another mechanism to be considered is called “the muscle tuning hypothesis” [28]. According to this hypothesis, most vibration damping occurs at the resonant frequencies of the tissues, concurring with the highest level of muscle activity. This suggests that body has a strategy to minimize its vibrations regardless of the mode of the input force. Forces that drive the soft tissues of lower extremity closer to resonance cause increases in muscle activity and damp the vibration transmission [28]. The resonant frequency of the tissues in leg is about 10 to 50 Hz [29, 30] and our applied frequency (30 Hz) was within that range so, increased strength in both muscles may be the result of muscle tuning hypothesis. Studies showed that in response to frequencies greater than 20 Hz, coupled rotational motions about the hip joint and greater muscle activation may also affect the transmission of vibratory-induced forces. Our findings are in agreement with those of Torvinen et al. They showed improvement in isometric limb extension strength after the application of whole body vibration (25–40 Hz, 3.6–6.4 g) for 4 months [31].

Trans and his colleagues investigated the effects of WBV (frequency = 25 Hz) on knee flexor and extensor muscles strength in patients with OA using isokinetic dynamometry. Their results confirmed that WBV can improve muscle strength [32].

Macahado and colleagues also found significant difference in maximum voluntary isometric contraction (MVIC) of knee and ankle extensors in WBV intervention group (10 weeks, frequency: 20–40 Hz, amplitude: 2 mm) in comparison to control group [33]. They attributed these changes to thigh muscle hypertrophy. In our study general muscle strength did not show significant difference statistically; subjects stood with 30° of knee flexion on vibrating plate and this position dampened the transmitted vibratory waves at hip level [29]; so there was no statistical significant difference between the groups in regard with general strength.

### Functional tests

#### TUGT

Significant difference between the two groups after 6 weeks indicated that the mobility and dynamic balance had improved in the intervention group. Our results are in line with those of Machado et al. who attributed the decrease of TUGT time to improvement in muscle mass [33].

Our results are also in accordance with those of Bruyere [34] and Bautmans [35]. An increase in body balance may explain the improvement in TUGT. Rogan and his colleagues evaluated the feasibility of WBV in a group of untrained elderly. The applied WBV frequency was 5 Hz, three times a week for 4 weeks. The expanded TUGT showed no significant changes albeit medium effect sizes [15].

UST is a measure for balance capabilities and a predictor for risk of fall. Although there was not significant improvement in UST in the intervention group, they showed increase in UST time after 6 weeks in comparison to control group who indicated decrease in UST time. Whether this 6-week time frame caused this decrement in time in the control group is not clear.

### Balance evaluation

Among several parameters of body sway derived from recordings of center of pressure (CoP) with the aid of a force platform, we evaluated mean velocity of CoP displacement (cm/s) that is considered to be the most valid parameter [36] because other derived parameters showed low levels in ICC tests. Also, four out of eight selected positions were performed completely by all participants. In the first position (eyes open, no foam), there was no significant difference between the groups after the study. This is an expected result because the subjects had mild to moderate neuropathy and had acceptable balance during standing. As the positions became more complicated, significant balance improvement had been observed in the intervention group. The position of standing on foam with eyes closed seems to be the hardest position among selected ones because in this position two of three main sources of balance and postural control systems are compromised; vision and somatosensory systems. The main effect of time was significant in this position. Therefore, apart from the effect of other parameters, mean velocity had significant difference before in comparison to after intervention. The interaction between time and group is also significant; so, the pattern of mean velocity changes is different between groups. Therefore, not only the mean velocity of the intervention group decreased, but also mean velocity of control group increased after 6 weeks.

Increased Ia afferents stimulation was considered as a possible neuromuscular mechanism for WBV but Abercromby’s study did not support this hypothesis [28]. Balance improvement can be attributable to postural control strategy that is adopted during WBV. As the knee angle of the subjects was small (30°) in our study and small knee angles are associated with a greater postural anxiety than are large knee angles, [28] the balance improvement may be mediated by the presence of a postural control mechanism.

Our study had some limitations. First is the lack to follow up. We cannot anticipate a timeframe during which the achieved improvements would remain. Second is that, we only evaluated 10 patients in each group which could have affected the power of the study. We could not design a double- blind study as well; so we propose an identical double- blind one to be performed. It would be better that if we had a sham group maintaining the same position on vibration plate for the same duration of time.

Our vibration plate was a synchronous type. There are some differences between plates vibrating vertically (VV) and the plates rotating around an anterior-posterior axis (RV). VVs may induce different degrees of muscle activation, postural challenge and tissue vibration in comparison to RVs [28]. We suggest that an identical study with RV machines be designed to compare the data with those of our study. Our study was the first to evaluate the effects of WBV intervention on diabetic patients with neuropathy. We propose that the future studies investigate other parameters to find the best training program for patients with diabetes. Our results indicated muscle strength enhancement in diabetic patients but we could not determine whether it was a neurogenic or a myogenic potentiation. Further studies with sonography or MRI should be performed to determine the exact mechanisms for strength enhancement. The exact mechanisms of neuromuscular enhancement during WBV are still unknown. Future studies of WBV are needed to examine the other underlying mechanisms.

## Conclusion

In conclusion, our results demonstrated that WBV with the frequency of 30 Hz and amplitude of 2 mm enhances the muscle isometric strength and improves the mobility and balance in type 2 diabetic patients with mild to moderate peripheral neuropathy. Also, applying WBV with these parameters is completely safe to be applied clinically for 2 diabetic patients with mild to moderate peripheral neuropathy.
